# Individuals at increased risk for development of bipolar disorder display structural alterations similar to people with manifest disease

**DOI:** 10.1038/s41398-021-01598-y

**Published:** 2021-09-20

**Authors:** Pavol Mikolas, Kyra Bröckel, Christoph Vogelbacher, Dirk K. Müller, Michael Marxen, Christina Berndt, Cathrin Sauer, Stine Jung, Juliane Hilde Fröhner, Andreas J. Fallgatter, Thomas Ethofer, Anne Rau, Tilo Kircher, Irina Falkenberg, Martin Lambert, Vivien Kraft, Karolina Leopold, Andreas Bechdolf, Andreas Reif, Silke Matura, Thomas Stamm, Felix Bermpohl, Jana Fiebig, Georg Juckel, Vera Flasbeck, Christoph U. Correll, Philipp Ritter, Michael Bauer, Andreas Jansen, Andrea Pfennig

**Affiliations:** 1 Department of Psychiatry and Psychotherapy, Carl Gustav Carus University Hospital, Technische Universität Dresden, Dresden, Germany; 2 Core-Facility Brainimaging, Faculty of Medicine, University of Marburg, Marburg, Germany; 3 Department of Psychiatry, University of Marburg, Marburg, Germany; 4 Center for Mind, Brain and Behavior (CMBB), University of Marburg and Justus Liebig University Giessen, Marburg, Germany; 5 Neuroimaging Center, Technische Universität Dresden, Dresden, Germany; 6 Institute for Medical Informatics and Biometry, Carl Gustav Carus Faculty of Medicine, Technische Universität Dresden, Dresden, Germany; 7 Department of Psychiatry and Psychotherapy, Tübingen Center for Mental Health, University of Tübingen, Tübingen, Germany; 8 Department for Biomedical Resonance, University of Tübingen, Tübingen, Germany; 9 Department of Psychiatry and Psychotherapy, University Medical Center Hamburg-Eppendorf, Hamburg, Germany; 10 Department of Psychiatry, Psychotherapy and Psychosomatic Medicine, Vivantes Hospital Am Urban and Vivantes Hospital Im Friedrichshain, Charité-Universitätsmedizin Berlin, Berlin, Germany; 11 Department of Psychiatry, Psychosomatic Medicine and Psychotherapy, University Hospital, Goethe University, Frankfurt, Germany; 12 Department of Psychiatry and Neurosciences, Charité Campus Mitte, Charité - Universitätsmedizin Berlin, Berlin, Germany; 13 Department of Clinical Psychiatry and Psychotherapy, Brandenburg Medical School Theodor Fontane, Neuruppin, Germany; 14 Department of Psychiatry, Psychotherapy and Preventive Medicine, LWL University Hospital, Ruhr-University, Bochum, Germany; 15 Department of Child and Adolescent Psychiatry, Charité Universitätsmedizin Berlin, Berlin, Germany; 16 Department of Psychiatry, Northwell Health, The Zucker Hillside Hospital, Glen Oaks, NY USA; 17 Department of Psychiatry and Molecular Medicine, Donald and Barbara Zucker School of Medicine at Hofstra/Northwell, Hempstead, NY USA

**Keywords:** Bipolar disorder, Diagnostic markers

## Abstract

In psychiatry, there has been a growing focus on identifying at-risk populations. For schizophrenia, these efforts have led to the development of early recognition and intervention measures. Despite a similar disease burden, the populations at risk of bipolar disorder have not been sufficiently characterized. Within the BipoLife consortium, we used magnetic resonance imaging (MRI) data from a multicenter study to assess structural gray matter alterations in *N* = 263 help-seeking individuals from seven study sites. We defined the risk using the EPI*bipolar* assessment tool as no-risk, low-risk, and high-risk and used a region-of-interest approach (ROI) based on the results of two large-scale multicenter studies of bipolar disorder by the ENIGMA working group. We detected significant differences in the thickness of the left pars opercularis (Cohen’s *d* = 0.47, *p* = 0.024) between groups. The cortex was significantly thinner in high-risk individuals compared to those in the no-risk group (*p* = 0.011). We detected no differences in the hippocampal volume. Exploratory analyses revealed no significant differences in other cortical or subcortical regions. The thinner cortex in help-seeking individuals at risk of bipolar disorder is in line with previous findings in patients with the established disorder and corresponds to the region of the highest effect size in the ENIGMA study of cortical alterations. Structural alterations in prefrontal cortex might be a trait marker of bipolar risk. This is the largest structural MRI study of help-seeking individuals at increased risk of bipolar disorder.

## Introduction

In recent years, there has been an increasing effort to define and characterize individuals at risk for psychiatric disorders. These efforts gave rise to specialized early recognition services, which provide risk-stratifications and targeted interventions for help-seeking individuals at risk [1, 2]. As an example, cumulative evidence from epidemiological, genetic, neuroimaging, and interventional studies led to the establishment of the psychosis risk syndrome, which is now included as a diagnostic category in the Diagnostic and Statistical Manual of Mental Disorders (DSM-5) [3]. Although bipolar disorder remains similarly prevalent and is associated with a disease burden comparable to psychotic disorders [4], the populations at risk have not been sufficiently identified and characterized [5].

Bipolar disorder has been associated with brain structural alterations. Whereas earlier structural magnetic resonance imaging (MRI) studies focused on cortical volume, more specific segmentation methods allowed for its discrete and developmentally distinct quotients—cortical thickness and surface area [6–8]. Up to date, the ENIGMA group performed two large-scale multicenter studies, which analyzed structural differences between individuals with bipolar disorder and healthy participants [9, 10], thereby overcoming typical limitations of single studies or traditional meta-analyses, such as low power, publication bias, or comparability of results [11]. In the first study, Hibar et al. [10]. investigated subcortical structures in 4304 participants and found reduced volumes of hippocampus, amygdala, and thalamus among individuals with bipolar disorder. In the second large-scale study, the authors [9] investigated cortical structures in a sample of 6503 participants and identified a pattern of significant reductions of cortical thickness in frontal, temporal, and parietal regions, with maximum effect size in the left inferior frontal gyrus—pars opercularis (see below). Moreover, the reductions of cortical thickness correlated with illness duration. This study detected no differences in the surface area. According to a recent meta-analysis, volumetric studies in patients in early stages of illness consistently reported reduced volume of the bilateral pregenual anterior cingulate cortex [12].

Structural MRI studies in people at risk for bipolar disorder have so far exclusively focused on genetic risk, i.e. studying the affected or unaffected first-degree relatives of bipolar patients. Interestingly, in contrast to patients with established disease, individuals with genetic risk seem to display rather increased cortical volume, particularly in the right inferior frontal gyrus, lingual gyrus, and superior temporal gyrus [13–15]. Higher cortical volume of the inferior frontal gyrus was proposed as a trait for genetic risk and may diminish as a result of disease progression or other contributing risk factors [14–16]. Regarding subcortical structures, there has been meta-analytical evidence for reduced amygdala volumes in adults with genetic risk while detecting no significant effects in other subcortical structures including hippocampus, striatum, and thalamus [13, 17].

Although a positive family history was shown to be the most robust risk factor for bipolar disorder, only a fraction of individuals with first-degree relatives will develop bipolar disorder (4.5−22.4%) [18–20], some may actually develop other disorders, such as major depression. Additionally, most bipolar patients do not have a reliable positive family history. Beyond the genetic risk, several non-genetic risk factors were suggested to be associated with an increased risk for bipolar disorder. These include specific subsyndromal manic symptoms, depressive syndromes, mood swings, changes in sleep and circadian rhythm, anxiety disorders, attention deficit hyperactivity disorder (ADHD), specific character traits, stressful life events, or substance abuse [5, 21]. Based on studies of these risk factors, several risk-assessment tools have been developed: Bipolar-at-risk (BAR) criteria [22], Extended BAR criteria (BARS) [23], Bipolar Prodrome Symptom Interview and Scale prospective version (BPSS-P) [24], Early Phase Inventory for Bipolar Disorders (EPI*bipolar*) [21] (see also Supplementary Table 1).

In contrast with traditional studies of first-degree relatives, in this study, we investigated structural alterations of the brain in *help-seeking* individuals at increased risk of bipolar disorders. This approach goes beyond the genetic risk approach to search for neural correlates of genetic, as well as *non*-genetic risk for bipolar disorder. We focused on the cortical thickness for the following reasons: 1. High-grade evidence for reduced cortical thickness in bipolar disorders in a large-scale multicenter study [9], 2. We hypothesized that the neural alterations in help-seeking individuals may represent a diverse pattern of factors beyond genetics which may be associated with structural alterations rather similar to those in patients with established disease (i.e. reduced cortical thickness).

In order to perform an analysis of cortical and subcortical structural alterations and simultaneously attain a sufficient statistical power, we performed a ROI approach (for details on the power calculation see Supplementary note 1). We based our choice on the two above-mentioned large-scale ENIGMA multicenter studies available at the time [9, 10]. Those studies detected cortical and subcortical differences between individuals with bipolar disorder and healthy controls, respectively, and published the lists of effect sizes for particular regions. From those data, we selected one cortical and one subcortical ROI based on the largest effect size in the given study. More specifically, we selected the left inferior frontal gyrus—pars opercularis (a region within the broader ventrolateral prefrontal cortex (VLPFC)) from Hibar et al. [9] and hippocampus from Hibar et al. [10]. As Hibar et al. [9] identified differences only in cortical thickness and not in the surface area, we analyzed the differences in cortical thickness between the bipolar risk groups for pars opercularis. As the above-mentioned modalities do not apply for subcortical structures, and in accordance with Hibar et al. [10]. We analyzed the differences in gray matter volume in the case of hippocampus. Lastly, we also aimed to detect possible widespread differences in cortical thickness or subcortical volumes in exploratory analyses outside the pre-specified ROIs. To our knowledge, the present study is the largest structural MRI study of help-seeking individuals at increased risk of bipolar disorder.

## Materials and methods

### Participants

In this study, we analyzed the MRI and baseline clinical data acquired in a subsample of the ongoing Early-BipoLife study. Details of the study protocol can be found elsewhere [25, 26]. Briefly, the Early-BipoLife study is a multicenter, naturalistic, prospective-longitudinal observational cohort study performed at ten German universities and teaching hospitals with early detection centers/facilities and specialized in- and outpatient care. Although Early-BipoLife is an ongoing study, the baseline acquisition of clinical and MRI data has been completed. Of the total *N* = 1 229 recruited adolescents and young adults (age 15−35) at risk, *N* = 313 volunteered to receive an MRI (recruited at seven of the ten study sites: Berlin, Bochum, Frankfurt, Hamburg, Dresden, Marburg, Tübingen) [27]. In order to include all proposed risk factors for bipolar disorder, we recruited the participants in three recruitment pathways: *N* = 123 were consulting early detection centers/facilities and were screened positive for an indication of ≥1 proposed risk factor for bipolar disorder, *N* = 146 were young in- and outpatients with a depressive syndrome and *N* = 44 had an established diagnosis of ADHD. For the complete inclusion criteria for each recruitment pathway see Supplementary Note 2. Participants were phenotyped in depth including the above-mentioned instruments to assess the risk for developing bipolar disorder. For the demographic characteristics of the sample, see Table 1 and Supplementary Tables 2, 3.

**Table 1:** Socio-demographic characteristics. In order to assess the risk-associated structural alterations in help-seeking individuals, we divided the participants into no-risk, low-risk, and high-risk groups using the EPI*bipolar* assessment tool.

	No-risk	Low-risk	High-risk	Test	Post hoc^a^
*N* (%) (*N*_total_ = 263)	32 (12.2)	130 (49.4)	101 (38.4)		
*Demographic*					
Sex female/male (%)	10/22 (31.3/68.8)	60/70 (46.2/53.8)	57/44 (56.4/43.6)	*χ2* = 6.642 *p* = 0.036*	No-risk ≠ high-risk; *χ2* = 6.166 *p* = 0.039*
Age (SD)	24.13 (3.08)	24.78 (4.55)	25.13 (4.42)	*F*(2,102.393) = 1.028 *p* = 0.361	
*Education*					
1. No degree/attending school (%)	0 (0.0)	8 (6.3)	4 (4.0)	*p* = 0.633^b^	
2. Secondary school (%)	6 (18.8)	18 (14.1)	18 (18.0)		
3. High school (%)	26 (81.3)	102 (79.7)	78 (78.0)		
*Recruitment pathway*					
1. Early recognition (%)	15 (46.9)	48 (36.9)	50 (49.5)		
2. Depression (%)	5 (15.6)	68 (52.3)	41 (40.6)		
3. ADHD (%)	12 (37.5)	14 (10.8)	10 (9.9)		
*Psychiatric medication*					
Yyes (%)	11 (34.4)	82 (63.6)	53 (53.0)	*χ2* = 9.432 *p* = 0.009**	No-risk ≠ low-risk; *χ2* *=* *8.955 p* = 0.009**
1. Antidepressants (%)	5 (15.6)	67 (51.5)	40 (39.6)		
2. Antipsychotics (%)	1 (3.1)	25 (19.2)	11 (10.9)		
3. Mood stabilizers (%)	0 (0.0)	7 (5.4)	4 (4.0)		
4. Anxiolytics & Sleep (%)	1 (3.1)	8 (6.2)	7 (6.9)		
5. Psychostimulants (%)	5 (15.6)	6 (4.6)	10 (9.9)		
*Substance use*					
Smoking status					
1. Never smoked (%)	18 (56.3)	63 (48.5)	39 (38.6)	*p* = 0.079^b^	
2. Current smoker (%)	9 (28.1)	59 (45.4)	52 (51.5)		
3. Past smoker (%)	5 (15.6)	8 (6.2)	10 (9.9)		
Cannabis present					
0. No use (%)	26 (81.3)	90 (69.2)	74 (73.3)	*p* = 0.830^b^	
1. <1×/month (%)	1 (3.1)	11 (8.5)	9 (8.9)		
2. ~1×/month (%)	0 (0.0)	8 (6.2)	6 (5.9)		
3. 2−9×/month (%)	2 (6.3)	8 (6.2)	6 (5.9)		
4. ≥10×/month (%)	3 (9.4)	13 (10.0)	6 (5.9)		
Cannabis lifetime					
0. No use (%)	15 (46.9)	48 (36.9)	42 (41.6)	*p* = 0.942^b^	
1. <1×/month (%)	8 (25.0)	28 (21.5)	19 (18.8)		
2. ~1×/month (%)	0 (0.0)	6 (4.6)	5 (5.0)		
3. 2−9×/month (%)	3 (9.4)	17 (13.1)	11 (10.9)		
4. ≥10×/month (%)	6 (18.8)	31 (23.8)	22 (21.8)		
*Genetic risk*					
First-degree relatives of BD patients *N* (%)	0 (0.0)	3 (2.3)	17 (16.8)		
Second-degree relatives of BD patients *N* (%)	0 (0.0)	0 (0.0)	8 (7.9)		

In order to assess the risk-associated structural alterations in help-seeking individuals, we divided the participants into no-risk, low-risk, and high-risk groups using the EPIbipolar assessment tool.

Abbreviations. *BAR-Criteria* Bipolar-at-Risk-Criteria; *BD* Bipolar Disorder.

^a^*p*-values have been adjusted using Bonferroni Correction.

^b^Fisher−Freeman−Halton’s exact test was used for variables with ≥1 expected cell counts <5. *p < 0.05; **p < 0.01; ***p < 0.001.

Exclusion criteria for study enrollment were as follows: diagnosis of bipolar disorder, schizoaffective disorder, schizophrenia; diagnosis of anxiety, obsessive-compulsive, or substance dependence disorder that fully explained the whole symptomatology; limited ability to comprehend the study; implied expressed negative declaration of intent to participate in the study by a minor; acute suicidality. The study was approved by the Ethics Committee of the Medical Faculty of the Technische Universität Dresden (No: EK290082014), as well as local ethics committees at each study site. We obtained a written informed consent after comprehensive information about study aims and procedures.

### Assessment of bipolar risk

We used the EPI*bipolar* interview for the primary analyses presented here. EPI*bipolar* integrates the broadest range of early and late risk factors for bipolar disorder [21]. EPI*bipolar* is a semi-structured interview, which classifies the participants according to the presence of main and secondary risk factors into no-risk, low-risk, and high-risk groups (for an overview see Supplementary Table 4). In this study, we used a modification of the original risk categories according to Leopold et al. [21], which accounts for the low sensitivity of the original high-risk category. In this analysis, the original high-risk and ultra-high risk groups were fused, as the high-risk group contained a disproportionally low number of participants (3.2%). EPI*bipolar* was primarily designed as a risk stratification tool for the purposes of targeted clinical intervention within early recognition services. From the clinical perspective, individuals with up to one secondary risk factor, or more secondary risk factors in a bipolar non-specific constellation might not benefit from a targeted intervention [21]. For this reason, these participants were assigned to the ‘no-risk’ category. The EPI*bipolar* interview is being further developed and currently undergoing a longitudinal validation within the BipoLife study [25, 26].

As there might be an overlap between bipolar risk and psychosis risk syndromes, we screened for the presence of a possible psychosis risk syndrome using PQ-16 self-report questionnaire [28]. In all participants scoring 6 or above, we evaluated psychosis risk status using the Structured Interview for Psychosis Risk Syndromes (SIPS) [29]_._

### MRI scanning

The BipoLife neuroimaging consortium involved seven study sites with different hardware and software configurations. At six sites, data were acquired at Siemens Magnetom MR scanners (Trio, Skyra, Prisma). One site had a Philips Achieva scanner. A detailed description of the scanning protocol (including details on participating study sites, MR scanners, specific hardware configurations) can be found in Vogelbacher et al. [27]. Pulse sequence parameters were standardized across all sites to the extent permitted by each platform.

All subjects were assessed with a large neuroimaging battery, involving both high-resolution structural T1-weighted images and functional measurements (for an overview, see Ritter et al. [26]). In the present study, we focused on the analysis of the T1-weighted MRI data. A detailed description of the pulse sequence parameters of all sites is given in Vogelbacher et al. [27].

For quality assurance, the MRI images were analyzed using the MRIQC tool [30]. MRIQC can qualify structural and functional MR images and highlights the results in a human-readable report. This report contains several metrics including a movement plot and a plot of the background noise. For this dataset, a visual inspection by two authors was performed. The focus of this inspection was the general quality of the data. A set of 23 subjects was excluded from further analysis due to strong movement, ghosting, or fold-over artifacts. A detailed description of the MRI quality control protocol is described elsewhere [27].

### MRI preprocessing

We used the freely available FreeSurfer 6.0 software to perform the cortical and subcortical segmentations [6, 31]. To speed up the preprocessing with FreeSurfer, the computation was conducted in parallel and distributed using the NICePype [32]. For the cortical parcellations, we used the Desikan-Killiany atlas [31, 33], obtaining cortical thickness values for 68 (34 left and 34 right) cortical regions. For the segmentation of subcortical regions, we used the standard volumetric segmentation atlas available in Freesurfer [29], obtaining gray matter volumes of 14 subcortical structures (7 left and 7 right).

Finally, we performed a standardized quality control of the cortical and subcortical segmentations and parcellations according to the established protocols of the ENIGMA working group (http://enigma.ini.usc.edu/protocols/imaging-protocols). Briefly, we visually inspected the segmented regions according to the internal and external surface methods and performed statistical outlier detection. The outliers were subjected for further visual inspection. We discarded those subjects, who did not pass the quality control or displayed major segmentation errors (*N* = 3). This number was rather low, potentially due to the preceding, thorough quality assessment using MRIQC (see above).

### Statistical analysis

We performed the primary, ROI-based statistical analyses using extracted cortical thickness values of the left pars opercularis and hippocampal volume in IBM SPSS Statistics (version 27). After quality assessment and discarding participants with missing values for the risk scores, *N* = 263 subjects were available for the statistical analysis. In our primary analysis, we analyzed structural differences between the three EPI*bipolar* risk groups (no-risk, low-risk, and high-risk) in two *á priori* selected ROIs— left pars opercularis and hippocampus (defined as bilateral mean hippocampal volume, see below) using generalized linear models. For the left pars opercularis, in accordance with prior ENIGMA study of cortical structures in bipolar disorder [9], we used cortical thickness as the dependent variable, while controlling for age, sex, current medication (yes/no), smoking status (current/past smoker/never smoked), lifetime and present (<6 months) cannabis use (for the detailed characteristics of variables and their dimensions see Table 1). We used the site as a random effect. The effect size for the significant result was calculated from partial eta squared according to Cohen [34]. For the hippocampus, in accordance with prior ENIGMA study of subcortical structures in bipolar disorder [10], we used the bilateral mean hippocampal volume as a dependent variable, while controlling for all above mentioned covariates/factors, as well as for the total estimated intracranial volume. We assessed the homogeneity of variances between the groups using the Levene test (*p* = 0.146, *p* = 0.145 for pars opercularis and hippocampus respectively). We corrected the *p*-values for two comparisons (for two ROIs analyzed) using the false discovery rate (FDR) correction [35].

We performed the following post hoc analyses: first, we tested for a linear effect of the risk on the cortical thickness. More specifically, we conducted a multiple regression analysis with bipolar risk, sex, current psychiatric medication, smoking status, present cannabis use, and study site as independent variables, and thickness of the left parsopercularis as the dependent variable. The bipolar risk was established as ordinale values 0, 1, and 2 corresponding to the EPI*bipolar* risk groups no-risk, low-risk, and high-risk respectively. We then continued to generate the regression equation considering all significant independent variables. To create the diagram showing the linear effect of EPI*bipolar* risk on the thickness of the left parsopercularis while holding the other independent variables constant, we inserted the sample means of the remaining significant independent variables (relative frequencies for dummy variables) into the regression equation and plotted the resulting equation (Supplementary Fig. 1). Second, we performed a whole-brain exploratory analysis using the Freesurfer Qdec tool available in FreeSurfer 6.0. We used a general linear model (GLM) to estimate the differences in cortical thickness at each vertex of the surface between the EPI*bipolar* risk groups. We used age and sex as covariates. We corrected the analysis for multiple comparisons using the FDR correction (*p* < 0.05). Third, we performed an exploratory analysis of the bilateral mean volumes of other subcortical structures (thalamus, amygdala, nucleus accumbens, caudate, globus pallidus, putamen) using generalized linear models as described above for hippocampal volume. The *p*-values were adjusted using FDR correction (*p* < 0.05). Finally, we repeated the primary ROI-based analyses of left pars opercularis and hippocampus using the risk defined by BARS and BPSS-P criteria (for details on the risk criteria see Supplementary Table 1). In accordance with the previous study [22], we used a modified variable representing the presence or absence of any BARS/BPSS-P criterion respectively (*N*_BAR__+_ = 186 (70.7%), *N*_BPSS-P+_ = 56 (21.3%)).

## Results

### ROI based primary analyses of left pars opercularis and hippocampus

**Fig. 1:**
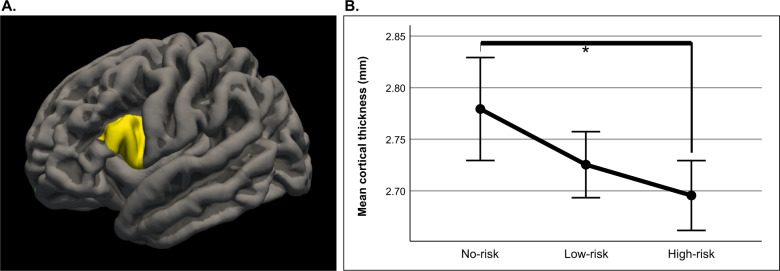
ROI-based analysis of cortical thickness by individuals at increased risk for development of bipolar disorder. **A** Left inferior frontal gyrus—pars opercularis as defined by the Desikan−Killiany atlas. **B** Mean thickness of the left pars opercularis. The post hoc tests revealed a significantly thinner cortex of the left pars opercularis between no-risk and high-risk groups. The low-risk group tends to have lower thickness than the no-risk group, while having a greater thickness than the high-risk group. However, these differences were not significant. ^*^ denotes statistical significance at *p* = 0.012 (FDR-corrected). Error bars represent the 95% confidence intervals.

We detected a significant difference in the mean thickness of the left pars opercularis [*F*(2, 245) = 4.475, *p* = 0.024 (FDR-corrected)] between the bipolar-risk groups (Fig. 1 and Table 2) with medium effect size (Cohen’s *d* = 0.47). Post-hoc tests revealed a significant difference between the no-risk and high-risk individuals (*p* = 0.012, FDR-corrected) (Fig. 1). The low-risk individuals displayed numerically thinner cortex of the left pars opercularis compared to the no-risk individuals as well, while showing a greater thickness than the high-risk individuals. However, both these pairwise comparisons were not statistically significant (*p* = 0.08 and *p* = 0.106 respectively, FDR-corrected).

**Table 2:** Results of the generalized linear models using the thickness of the left pars opercularis as an independent variable.

Measure	*F*	*p*-value	Partial Eta squared
EPI*bipolar* risk	4.475	0.012	0.035
Sex	1.417	0.235	0.006
Smoking status	0.336	0.715	0.003
Medication	2.171	0.142	0.009
Site	3.489	0.002	0.079
Age	9.152	0.003	0.036
Cannabis present	1.069	0.302	0.004
Cannabis lifetime	0.106	0.745	0.000

The significance test displays uncorrected *p*-values.

We detected no significant difference in the mean volume of hippocampus [*F*(2, 244) = 0.640, *p* = 0.528 (FDR-corrected)] between the bipolar-risk groups (Table 3).

**Table 3:** Results of the generalized linear models using the hippocampal volume as an independent variable.

Measure	*F*	*p*-value	Partial Eta squared
EPI*bipolar* risk	0.640	0.528	0.005
Sex	9.821	0.002	0.039
Smoking status	0.677	0.509	0.006
Medication	1.335	0.249	0.005
Site	11.566	0.000	0.221
Age	0.947	0.332	0.004
Cannabis present	0.094	0.760	0.000
Cannabis lifetime	0.197	0.868	0.004
ICV	108.688	0.000	0.308

The significance test displays uncorrected *p*-values. ICV—estimated total intracranial volume.

### Secondary regression analysis of left pars opercularis

A post hoc analysis using a multiple regression revealed a significant regression equation (*F*(13, 249) = 3.502, *p* < 0.001, *R*^2^ = 0.16) with a linear effect of the risk score on the thickness of the pars opercularis (*β* = −0.037, *p* = 0.004) (Supplementary Fig. 1).

### Secondary ROI based analysis using BARS and BPSS-P criteria

There were no significant differences between the participants fulfilling vs. not fulfilling any BARS criterion in the thickness of pars opercularis [*F*(1, 247) = 1.681, *p* = 0.196] or hippocampal volume [*F*(1, 246) = 0.082, *p* = 0.775]. There were no significant differences between the participants fulfilling vs. not fulfilling any BPSS-P criterion in the thickness of pars opercularis [*F*(1, 246) = 3.289, *p* = 0.071] or hippocampal volume [*F*(1, 245) = 0.710, *p* = 0.4].

### Exploratory analyses

The whole-brain exploratory analysis of cortical thickness showed no significant clusters displaying differences between the risk groups (FDR-corrected). The exploratory analysis of the volumes of the subcortical structures showed no significant differences in volumes (FDR-corrected): amygdala [*F*(2, 244) = 0.721, *p* = 0.487], caudate [*F*(2, 244) = 1.135, *p* = 0.485], putamen [*F*(2, 244) = 1.727, *p* = 0.36], nucleus accumbens [*F*(2, 244) = 4.439, *p* = 0.078], thalamus [*F*(2, 244) = 1.914, *p* = 0.36], pallidum [*F*(2, 246) = 0.875, *p* = 0.487].

## Discussion

In this study of help-seeking individuals at risk of bipolar disorder, we detected a significantly thinner left inferior frontal gyrus—pars opercularis in the high-risk individuals compared to the no-risk individuals. Moreover, there was a negative trend in the low-risk individuals. A post hoc analysis revealed an inverse linear effect of the risk group on the thickness of the left pars opercularis. There were no differences in the cortical thickness in the whole-brain exploratory analysis. We either did not detect any differences in the volume of the hippocampus and other subcortical regions.

In accordance with the studies of established bipolar disorder, this study reports thinner cortex in help-seeking individuals at risk of bipolar disorder. A previous, large-scale multicenter study detected a pattern of reduced thickness of frontal, temporal, and parietal cortex [9]. The significantly thinner cortex of the left pars opercularis in high-risk individuals in our study corresponds to the region of reduced thickness with maximum effect size in the patients of established disease in Hibar et al. [9]. Other multicenter studies detected a similar pattern of reduced cortical thickness in patients with schizophrenia, but not in other disorders, such as major depression, ADHD, obsessive-compulsive disorder, or autism [11, 36]. This suggests, that individuals at risk of bipolar disorder display structural alterations similar to the bipolar/schizophrenia spectrum.

Structural and genetic similarities between bipolar disorders and schizophrenia are well known [11, 36, 37]. The symptoms of psychosis prodrome might also overlap with bipolar prodrome [38]. However, in our sample, we specifically recruited participants with bipolar risk factors, while manifest psychotic disorders were an exclusion criterion. Of all included participants, only six (2.3%) fulfilled the psychosis-risk status using the SIPS assessment tool. For this reason, we do not consider psychosis risk being a cofounder in our analysis.

Our findings of the thinner cortex are in contrast with previous studies of individuals at risk for bipolar disorder, which focused exclusively on affected and/or non-affected first-degree relatives of bipolar patients. These identified possible structural biomarkers of genetic risk, such as increased volume of the right inferior frontal gyrus, superior temporal gyrus, or increased intracranial volume [13–16]. At the same time, significantly more participants with genetic risk were assigned into the high-risk category in our sample. This might be counterintuitive, however, from the neuroimaging point of view, the early markers of a genetic risk for bipolar disorder seem to follow a trajectory towards reduction of volume/thickness, as the prodromal state or the disease progress. The above-mentioned findings of increased cortical volume are the likely biomarkers of genetic risk, especially before the manifestation of psychiatric symptoms. During the disease progression, other, non-genetic factors might override those early markers. The volume of the right inferior frontal gyrus was the largest in non-affected participants with genetic risk, and correlated inversely with illness duration in a combined group of affected participants with genetic risk and young subjects with bipolar disorder [15]. Interestingly, in a machine learning study using structural MRI, non-affected individuals with genetic risk were more accurately differentiated from healthy controls than individuals with genetic risk who already displayed clinical symptoms [39]. Finally, large-scale analysis suggests rather a widespread reduction of cortical thickness in manifest bipolar disorder [9, 11]. In order to study a comprehensive composition of risk factors beyond the genetic risk, the Early-BipoLife study included young help-seeking individuals with symptoms. For this reason, in our primary hypothesis, we expected structural alterations more typical for a disease progression, rather than for genetic risk. Although we did not test for differences in cortical volume, our finding of reduced cortical thickness in the left inferior frontal gyrus (pars opercularis) makes a potential finding of increased volume less likely. For this reason, we conclude, that we do not observe structural alterations directly associated with genetic risk in our analysis.

We detected a negative linear relationship between risk category and cortical thickness of the left pars opercularis. This suggests, that the cortical thickness is associated with the total number of risk factors, i.e. the magnitude of the risk. Cortical thickness was shown to decrease with age in a linear manner in young individuals, while different genes may contribute to cortical change in different ages [8, 16]. This phenomenon might be locally accelerated in individuals at risk for bipolar disorders. Longitudinal, structural MRI, and genetic analyses are necessary to assess the dynamic changes in individuals at risk.

In our statistical model, the effect size of the thinner cortex was moderate (as defined by Cohen’s *d* of 0.47). This is higher than the effect sizes in other above-mentioned multicenter studies of patients with established disease (Cohen’s *d* ≤ 0.293) [9, 10]. Compared to the multicenter studies mentioned, here we present a sample of adolescents and young adults in the early stages of the potential development of bipolar disorder with a low cumulative dose of medication. Whereas those studies pooled datasets acquired within independent single studies, the recruitment and data acquisition in our study was performed in each center according to the same study protocol. These factors might have contributed to the effect size. As the expected effect sizes were small, we performed a literature-based, hypothesis-driven ROI analysis. There might be other affected regions as well (for example the widespread prefrontal or temporal cortices), however, our sample would not have significant power to detect weak effects in a whole-brain exploratory analysis.

Collecting data using the same study protocol for each center under close supervision enabled us to control for diverse factors, which might contribute to differences in cortical thickness. Beyond age, sex, and current medication, we also controlled for smoking status, past and present cannabis use. The cortical thickness reduction of pars opercularis stayed significant even with correcting for these confounders. We did not correct for education status, as the majority of individuals in all groups attended high-school. Correcting for cannabis use might be misleading, as substance abuse is one of the risk factors in EPI*bipolar*. However, here the corresponding risk-defining item is not equivalent, as it evaluates increasing, periodic substance abuse, which is only one aspect of possible patterns of cannabis use. This criterion was met only in a few (1.5%) subjects in our sample. On the other hand, chronic cannabis use was associated with patterns of cortical thickness reductions also in prefrontal regions, including left pars opercularis [40–42]. For that reason, correcting for cannabis use in this structural MRI study is still appropriate.

A considerable amount of individuals in our sample had Axis I diagnoses other than bipolar disorder. We also intentionally included young patients with diagnosed depression and/or ADHD. As these have been associated with increased risk for bipolar disorders, pooling these participants is an appropriate way to study bipolar risk. This is also in line with previous MRI studies which analyzed affected participants with genetic risk, who met the criteria for mood disorders [15]. As a result of the population design of this study, the resulting risk groups were not matched according to the recruitment pathway, resulting in more participants with diagnosed depression in the low-risk and the high-risk group. On the other hand, there were no significant reductions of cortical thickness in the VLPFC/inferior frontal gyrus in major depression according to the ENIGMA working group [11, 43]. Patients with ADHD displayed reduction of average cortical thickness [11, 44], however, in our sample there were no differences in the number of participants recruited over the ADHD recruitment pathway between the risk groups.

Interestingly, in a secondary analysis, we did not detect differences in cortical thickness of pars opercularis or hippocampal volume between individuals meeting and not meeting any BARS criterion. However, in our sample, the BARS positive group consisted almost exclusively of participants who fulfilled the criterion for depressive symptoms. According to a recent multicenter study, there was no involvement of VLPFC alterations in major depression [43]. However, several studies described differences in hippocampal volume [45, 46]. On the other hand, these might be missing in young individuals without recurrent episodes, who are predominantly represented in our sample [47]. Similarly, we did not detect any significant structural differences using BPSS-P. Interestingly, the *p*-value for pars opercularis was rather small, which might indicate a statistical trend towards reduction. However, in this case, only 21.3% of participants fulfilled the criterion for any risk state.

The mean age of individuals in our sample was higher than the typical onset of bipolar disorder (24.84 (SD = 4.4)), which might suggest, that we were looking at individuals with a degree of resilience. Approximately 70% of all individuals would develop bipolar disorder by the age of 21 [48, 49]. However, the time-to-diagnosis by bipolar disorder is typically long. Depending on the structure of psychiatric services, it might take 8.7−12.4 years from the appearance of the first symptoms to establish the diagnosis [47–50]. Due to the predominance of depressive symptoms, as well as difficulties to recognize hypomania, the most typical false diagnosis is unipolar depression [24, 48, 49]. For this reason, we cannot make conclusions on resilience. On the other hand, we can assume that some individuals with depression in our sample might have an unrecognized bipolar disorder.

Inferior frontal gyrus has been implicated as an important functional hub in emotion regulation and cognitive control. Several studies detected aberrant functional activation and/or functional disconnection of this region. Task-based fMRI studies showed lower activation of the inferior frontal cortex, particularly in response to emotionally salient stimuli in youth at high risk of bipolar disorder [51]. Functional disconnection of the inferior frontal gyrus was detected in young individuals with bipolar disorder using resting-state fMRI [52]. From the functional point of view, an impaired inhibitory function of the inferior frontal cortex, may represent a trait marker of vulnerability to bipolar disorder [51, 53].

Functionally, cortical thickness reflects the volume of cells in a cortical column, the basic cortical functional unit [16]. Most of the anatomical connectivity takes place within cortical columns, rather than amid brain regions. Cortical thickness was also shown to be more dependent on local or intrinsic factors, rather than on the input from subcortical structures [8]. Our results, therefore, suggest, that the reduced cortical thickness in VLPFC/pars opercularis might be an early trait marker of risk for bipolar disorders, rather than a secondary effect of structural alterations in other, functionally connected (for example subcortical) structures.

Our work has several limitations. We used the risk for bipolar disorder according to the EPI*bipolar* interview for our analyses. We do not know yet, which participants will in the future develop bipolar disorder. However, the composition of risk factors giving rise to the risk categories was based on state-of-the-art evidence in the field. We also used other instruments (BARS, BPSS-P) with better validated psychometric properties; however, the validation studies lack longitudinal validation (BPSS-P) [24, 54], were performed in small samples (BAR), or lack replication (BARS) [22]. However, as the early recognition of bipolar disorder is an emerging field, the risk assessment tools are being further developed [25] (Supplementary Table 1). In our secondary analysis, there was a discrepancy between the statistically significant results among the risk assessment instruments. Future studies with more power are necessary, to evaluate the reproducibility of significant structural findings among the various risk instruments.

As this was a naturalistic, population-based observational study, a control group was not included. As a result, most of the participants displayed at least minor symptoms. Analyzing population-based samples using severity subgroups is a reasonable approach, which may bring relevant information [55]. Including control groups in future studies is essential to determine the specificity of the findings. Although the generalized linear models approach is suitable for analyses of unequal groups, the resulting sample size of the no-risk group was relatively small for a neuroimaging study.

In summary, we detected structural alterations similar to patients with manifest bipolar disorder, which correlate with the amount of known risk-factors. We detected reduced cortical thickness in a region, which has been functionally implicated as a potential biomarker of bipolar risk [51, 53]. Subcortical structures might not be structurally affected. Our secondary analyses, as well as large-scale multicenter studies of psychiatric disorders, do not suggest, that our finding of the thinner cortex was due to another diagnosis.

As this is an emerging field, the concept of the risk for bipolar disorders is being further developed. We based the risk definition on the presence of known risk factors according to a systematic literature review. Longitudinal studies, as well as studies including healthy controls, are necessary, to evaluate the exact predictive validity and specificity of our findings. An interesting approach for future studies would be to include patients with psychosis risk, in order to identify specific structural differences between risk syndromes displaying possibly overlapping structural differences. Machine learning studies may provide a tool to extract the information from cortical thickness into individual risk-stratification and/or risk-prediction tools [9, 56, 57].

## Supplementary information

Supplemental information
