# Supplementary material for: Individuals at increased risk for development of bipolar disorder display structural alterations similar to people with manifest disease
Source: Transl Psychiatry. 2021 Sep 20;11:485. doi: 10.1038/s41398-021-01598-y (PMC8452775; doi:10.1038/s41398-021-01598-y)

Individuals at increased risk for development of bipolar disorder display structural alterations similar to people with manifest disease

Supplementary materials

Supplementary Note 1: Power analysis

Supplementary Note 2. Inclusion and exclusion criteria for the Early-BipoLife study.

Supplementary Table 1. Overview of instruments for the bipolar risk assessment tools.

Supplementary Table 2. Breakdown of demographic characteristics per study site.

Supplementary Table 3. Breakdown of lithium intake per recruitment pathway.

Supplementary Table 4. EPI*bipolar* risk criteria.

Supplementary Figure 1. Partial plot for the linear effect of EPI*bipolar* risk on thickness of the left pars opercularis.

Supplementary Note 1: Power analysis

We performed the power estimation in performed in G*Power v.3.1.9.4. The target N = 310 would achieve a power of 70% and an alpha-error probability of 0.075. The calculation was based on the estimated effect sizes in prior studies: left pars opercularis Cohen's d = -.293 [9], hippocampus Cohen's d = -.232 [10].

Supplementary Note 2. Inclusion and exclusion criteria for the Early-BipoLife study.

Inclusion criteria

In order to include all proposed risk factors for bipolar disorder, we recruited the participants in three recruitment pathways[1].

1. Help-seeking persons:

• Age: 15 to 35 years

• Consultation of an early recognition centre/facility

• Presence of at least one of the proposed risk factors for bipolar disorder: Family history of bipolar disorder, (sub)threshold affective symptomatology/depressive syndrome, hypomanic/mood swings, disturbances of circadian rhythm/sleep other clinical hints

2. Young individuals with diagnosed depression:

• Age: 15 to 35 years

• In- or outpatients with a depressive syndrome in the context of: Major depressive disorder, dysthymic disorder, cyclothymic disorder, minor depressive disorder, recurrent brief depressive disorder, adjustment disorder with depressed mood, depressive disorder Not Otherwise Specified (NOS)

3. Patients with ADHD:

• Age: 15 to 35 years

• In- or outpatients with a clinically confirmed ADHD diagnosis

Exclusion criteria:

• Diagnosis of: bipolar disorder, schizoaffective disorder, schizophrenia

• Diagnosis of anxiety, obsessive–compulsive or substance

dependence disorder that fully explains the whole symptomatology

• Limited ability to comprehend the study

• Implied expressed negative declaration of intent to participate

in the study by a minor and

• Acute suicidality

Supplementary Table 1. Overview of instruments for the bipolar risk assessment tools (adapted according to Bröckel et al.).

**Instrument**	**Risk states**	**N (% Sample)**	**Validation**	**Note**
BPSS-P	Attenuated mania symptom syndrome (AMSS)	54 (20.5)	Good internal consistency, convergent validity and inter-rater reliability[2]	Semi-structured interview based on the DSM-5 criteria for bipolar disorder and major depressive disorder[2]
	Genetic mania risk and deterioration syndrome (GMRDS)	2 (0.8)		
BAR criteria	Sub-threshold mania, assessed by BPSS-P	26 (9.9)	Increased risk of developing bipolar disorders (HR = 5.30), poor prognostic accuracy (Harrell's C = 0.659)[3]	Ultra-high-risk criteria for BD[4]
	Sub-threshold depression, assessed by BPSS-FP or SCID *and* cyclothymic features	148 (56.3)		
	Sub-threshold depression plus genetic risk	13 (4.9)		
Extended BAR criteria (BARS)	Mixed symptoms, assessed by BPSS-P	3 (1.1)	BARS criteria had an adequate prognostic accuracy (Harrell's C = 0.742) and clinical utility[3]	Extension of the BAR criteria (2 additional symptom domains)[3]
	Mood swings, assessed by EPI*bipolar*	117 (44.5)		
EPI*bipolar*	No-risk	32 (12.2)	No longitudinal (ongoing study)<br><br>Includes and integrates items from validated tools (BPSS-P, BAR) as well as genetic risk	Semi-structured interview<br><br>Integrates risk factors based on a systematic review of literature[5]
	Low-risk	130 (49.4)		
	High-risk	101 (38.4)		

Supplementary Table 2. Breakdown of demographic characteristics per study site.

Site	N- total	N - female (%)	Mean age (SD)	N – No-risk (%)	N – Low-risk (%)	N – High-risk (%)
Dresden	36 (13.7)	23 (63.9)	25.19 (3.640)	6 (16.7)	19 (52.8)	11 (30.6)
Marburg	70 (26.6)	28 (40)	24.11 (3.740)	9 (12.9)	34 (48.6)	27 (38.6)
Frankfurt	39 (14.8)	21 (53.8)	26.13 (4.697)	7 (17.9)	16 (41)	16 (41)
Berlin	59 (22.4)	27 (45.8)	24.71 (4.190)	5 (8.5)	30 (50.8)	24 (40.7)
Tuebingen	19 (7.5)	9 (47.4)	25.63 (3.639)	3 (15.8)	8 (42.1)	8 (42.1)
Bochum	8 (3.0)	4 (50)	28.0 (4.504)	2 (25.0)	5 (62.5)	1 (12.5)
Hamburg	32 (12.2)	15 (46.9)	23.41 (5.724)	0 (0)	18 (56.3)	14 (43.8)

Supplementary Table 3. Breakdown of lithium intake per recruitment pathway.

**Recruitment pathway**	**Mood stabilizers N (%)**	**Lithium N (%)**
Help-seeking persons	4 (3.5)	3 (2.5)
Young individuals with diagnosed depression	6 (5.3)	4 (3.5)
Patients with ADHD	1 (2.8)	0 (0)

Supplementary Table 4. EPI*bipolar* risk criteria[5]. Adopted according to Bröckel et al. (in submission). The former ultra high-risk and high-risk groups were fused. The former intermediate-risk group was renamed as low-risk.

<table>
<tr><th>Main risk factors</th><th>Secondary risk factors</th><th>Risk groups</th></tr>
<tr><td rowspan="3">• Family history of bipolar disorder
• Increasing cyclothymic mood swings with change of activity
• Subtreshold manic symptoms</td><td>Group A

• specific disturbances in sleep and/or circadian rhythm
• increasing cyclothymic mood swings without change of activity
• specific depressive features</td><td>No-risk:

• none risk constellations mentioned below are met</td></tr>
<tr><td rowspan="2">Group B

• positive family history for MDD, schizoaffective disorder or schizophrenia (not applicable if genetic vulnerability for bipolar disorder is a main risk factor)
• any affective disorder lifetime
• lifetime and present ADHD or conduct disorder
• impairment in psychosocial functioning
• specific substance misuse
• episodic course of symptoms</td><td>Low-risk:

• one or more risk factors of group A and one or more risk factors of group B, without any main risk-factor
• family history of bipolar disorder as main factor, without any other risk factors</td></tr>
<tr><td>High-risk:

• one main risk factors and one or more secondary risk-factors of group A and/or group B are met
• or more than one main risk factor</td></tr>
</table>

Supplementary Figure 1. Partial plot for the linear effect of EPI*bipolar* risk on thickness of the left pars opercularis. To create the diagram showing the linear effect of EPI*bipolar* risk on thickness of the left parsopercularis while holding the other independent variables constant, we plotted cortical thickness minus all other regression components as a function of the risk score.

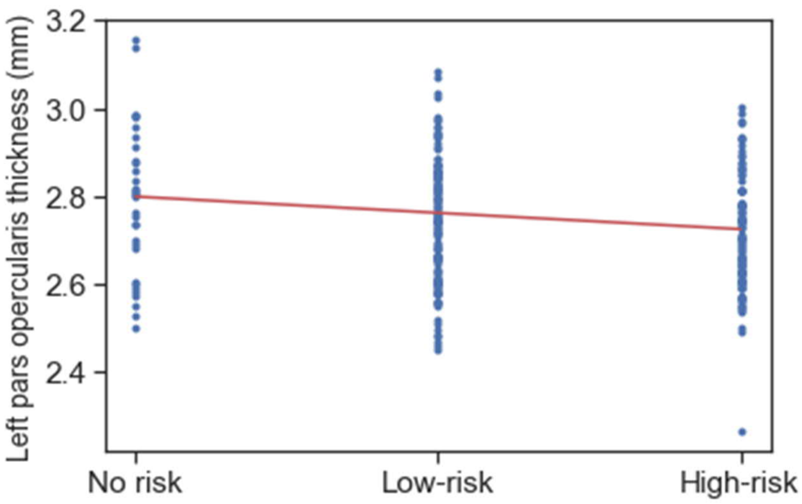

Supplement: Supplementary file 1 — Supplemental information [file 41398_2021_1598_MOESM1_ESM.pdf]
